# Prospects for NK Cell Therapy of Sarcoma

**DOI:** 10.3390/cancers12123719

**Published:** 2020-12-11

**Authors:** Mieszko Lachota, Marianna Vincenti, Magdalena Winiarska, Kjetil Boye, Radosław Zagożdżon, Karl-Johan Malmberg

**Affiliations:** 1 Department of Clinical Immunology, Doctoral School, Medical University of Warsaw, 02-006 Warsaw, Poland; mieszko.lachota@wum.edu.pl; 2 Department of Cancer Immunology, Institute for Cancer Research, Oslo University Hospital, 0310 Oslo, Norway; marianna.vincenti@rr-research.no; 3 Department of Immunology, Medical University of Warsaw, 02-097 Warsaw, Poland; mwiniarska@wum.edu.pl; 4 Department of Oncology, Oslo University Hospital, 0310 Oslo, Norway; kjetil.boye@rr-research.no; 5 Department of Clinical Immunology, Medical University of Warsaw, 02-006 Warsaw, Poland; 6 Center for Infectious Medicine, Department of Medicine Huddinge, Karolinska Institutet, Karolinska University Hospital, 141 86 Stockholm, Sweden

**Keywords:** Natural Killer (NK) cells, immunotherapy, sarcoma, cancer, chimeric antigen receptor (CAR), adoptive cell therapy, tumor microenvironment (TME), cell-mediated cytotoxicity, solid tumors

## Abstract

**Simple Summary:**

Sarcomas are a group of aggressive tumors originating from mesenchymal tissues. Patients with advanced disease have poor prognosis due to the ineffectiveness of current treatment protocols. A subset of lymphocytes called natural killer (NK) cells is capable of effective surveillance and clearance of sarcomas, constituting a promising tool for immunotherapeutic treatment. However, sarcomas can cause impairment in NK cell function, associated with enhanced tumor growth and dissemination. In this review, we discuss the molecular mechanisms of sarcoma-mediated suppression of NK cells and their implications for the design of novel NK cell-based immunotherapies against sarcoma.

**Abstract:**

Natural killer (NK) cells are innate lymphoid cells with potent antitumor activity. One of the most NK cell cytotoxicity-sensitive tumor types is sarcoma, an aggressive mesenchyme-derived neoplasm. While a combination of radical surgery and radio- and chemotherapy can successfully control local disease, patients with advanced sarcomas remain refractory to current treatment regimens, calling for novel therapeutic strategies. There is accumulating evidence for NK cell-mediated immunosurveillance of sarcoma cells during all stages of the disease, highlighting the potential of using NK cells as a therapeutic tool. However, sarcomas display multiple immunoevasion mechanisms that can suppress NK cell function leading to an uncontrolled tumor outgrowth. Here, we review the current evidence for NK cells’ role in immune surveillance of sarcoma during disease initiation, promotion, progression, and metastasis, as well as the molecular mechanisms behind sarcoma-mediated NK cell suppression. Further, we apply this basic understanding of NK–sarcoma crosstalk in order to identify and summarize the most promising candidates for NK cell-based sarcoma immunotherapy.

## 1. Introduction

Natural killer (NK) cells are the first-discovered members of the innate lymphoid cell (ILC) family, providing defense against tumors and pathogen-infected cells [1,2]. They express a remarkably diverse repertoire of inhibitory and activating surface receptors, regulating their responses [3]. NK cell-activating receptors recognize either stress-induced ligands, virus-encoded proteins, or Ig-coated cells. In contrast, inhibitory receptors contribute to self/non-self-discrimination by recognizing polymorphic major histocompatibility complex (MHC) class I ligands, also known as human leukocyte antigen (HLA) (Table 1) [3,4,5]. 

Different families of NK cell-activating receptors include NK Group 2 (NKG2) receptors, natural cytotoxicity receptors (NCRs), DNAM-1, 2B4, and CD16 (FcRyIIIa). NKG2D is an activating receptor belonging to the NKG2 family, recognizing MHC class I-related chain A/B (MICA/B) and members of the UL-16 binding protein (ULBP) family [6,7]. The NCRs include NKp30, NKp44, NKp46, and NKp80, which bind to B7-H6, AICL, or viral hemagglutinins [8,9,10]. DNAM-1 recognizes the viral receptors PVR (CD155) and Nectin-2 (CD112). 2B4 binds other SLAM family proteins, whereas CD16 is an Fc receptor for IgG, responsible for mediating antibody-dependent cell cytotoxicity (ADCC) against antibody-opsonized cells [11,12,13]. The primary NK cell inhibitory receptors are the NKG2A and the long-tailed killer cell immunoglobulin-like receptors (KIRs), which both bind to MHC class I molecules, preventing NK-mediated lysis of cells with normal MHC expression [14]. Inhibitory KIRs are specific for different MHC isotypes [14]. Acquisition of self-MHC class I binding KIRs during cell differentiation tunes NK cells’ cytotoxic potential in a process termed education. Repeated interactions of inhibitory KIRs with self-MHC class I molecules allow NK cells to acquire superior cytotoxic properties as well as tolerance to self-MHC expressing cells [15,16,17]. The dynamic functional tuning of human NK cells during NK cell differentiation and education and the implications for NK cell therapy are discussed in detail in [18].

As their name implies, NK cells can kill transformed or infected cells without the need for earlier priming. Their cytotoxicity is executed either by degranulation, where the directional release of perforin and granzymes induces apoptosis predominantly in a caspase-3-dependent manner, or through death receptor ligands of the tumor necrosis factor (TNF) family, such as TNF, TNF-related apoptosis-inducing ligand (TRAIL), and Fas ligand (FasL), acting primarily through caspase-8 [19,20]. Additionally, NK cells rapidly produce chemokines and cytokines upon activation, including interferon (IFN)-γ, granulocyte-macrophage colony-stimulating factor (GM-CSF), interleukin (IL)-10, CCL3, CCL4, CCL5, and CXCL8 that recruit and affect the function of hematopoietic and nonhematopoietic cells in the tumor microenvironment (TME) [21]. 

A growing amount of evidence suggests that the proper functioning of NK cells plays a significant role in immune surveillance of cancer, prompting researchers to utilize NK cells in cancer treatment [22,23]. Since NK cells do not express rearranged antigen receptors, they can be easily transferred across MHC barriers without causing graft-versus-host disease (GvHD). Lack of MHC restriction and their unique ability of cancer cell recognition through interactions of multiple surface molecules impedes cancer immune evasion by MHC downregulation or a single antigen loss [24]. Because of these potent antitumor properties, intense studies are currently being carried out to use NK cells, induced pluripotent stem cell (iPSC)-derived NK cells, and the NK cell line NK-92 as novel therapeutic tools against cancer [25,26,27]. However, NK cell therapy faces many challenges, such as inadequate homing properties, hostile TME, or tumor immunoevasion [28]. 

Notably, one of the most NK cell-sensitive cancer types are sarcomas, a heterogeneous group of aggressive mesenchyme-derived tumors with poor prognosis [29,30,31,32]. Sarcomas can originate from different tissues such as bone, cartilage, muscle, adipose tissue, or blood vessels. Sarcoma’s yearly incidence is approximately 5 per 100,000, accounting for less than 1% of malignant solid tumors in adults but more than 20% in children [33,34]. The mainstay of sarcoma treatment based on a combination of surgery and radiotherapy (RT) is able to control localized tumors; however, ~40% of the patients experience tumor relapse and distant metastases [29,35]. Unfortunately, current treatment regimens are ineffective in increasing overall survival in metastatic sarcomas, ranging from 11 to 20 months, creating a demand for novel and effective therapies [35,36]. The urgent nature of the demand is further underlined by the fact that irrespectively of the stage, some sarcoma subtypes have very few lines of systemic therapy with a clinically meaningful effect [35]. Both experimental and clinical data support the immune system’s involvement in sarcoma tumorigenesis. Spontaneous regressions and efficient immunosurveillance are observed in sarcomas, suggesting the prime role of the immune system in tumor development and prompting researchers to explore the potential use of immunotherapies in sarcoma treatment [22,37,38,39].

Sarcomas are the first type of cancer for which immunotherapy was effectively applied. William B. Coley injected streptococcal organisms into the tumors, based on observations of tumor regressions in patients with concomitant streptococcal infections in the last decade of the 19th century. More than half of the inoperable sarcoma patients treated by Coley were reported to respond completely [40]. Unfortunately, due to poorly characterized preparation and unpredictable toxicities, “Coley′s toxins” never became clinically useful.

Because of the unique relationship between NK cells and sarcomas, we set out to review the intimate crosstalk between NK cells and sarcoma cells during tumor initiation, promotion, progression, and metastasis. Further, we discuss the current knowledge regarding sarcoma immunoevasion and NK cell functionality. Finally, we review NK cell-based therapeutic approaches in sarcoma treatment, tested in both preclinical and clinical settings.

## 2. NK Cell Immune Surveillance during Distinct Phases of Sarcoma Development

NK cell-mediated immunosurveillance is an important factor in cancer development, especially in metastasis control. Since sarcomas have been identified as one of the most NK-sensitive solid tumors, they are a well-suited model to study NK cell-mediated cancer surveillance and tumor immunoediting [30]. Different combinations of NK cell-activating ligands such as MICA/B ULBP1/2/3/5, CD155, and CD112 are known to be expressed on both primary sarcoma samples and cell lines, allowing for NK cell cytotoxicity [41,42,43,44,45]. The primary pathway of killing sarcoma cells appears to be granule-dependent, with FasL-Fas interactions playing a minor role, possibly due to acquired FasL resistance [43,46,47]. However, the significance of the FasL-Fas pathway may be underestimated because of technical limitations. A 4-h incubation time during a standard in vitro cytotoxicity assay is insufficient to study death receptor-mediated apoptosis [48,49]. Indeed, NK cells were recently shown to kill target cells by both mechanisms in a sequential manner, starting with granzyme B-dependent killing and then gradually transitioning to death-receptor killing during serial killing events [50].

A decrease in NK cell cytotoxicity in older adults is associated with an increased risk of cancer development [51]. Further, pediatric osteosarcoma (OS) patients have a decreased number of circulating NKs, together implicating NK cells′ potential role in controlling tumor initiation and progression [52]. Sarcomas have a scarce immune infiltration compared to other solid tumors [53]. Current evidence of the prognostic role of lymphocyte infiltration in sarcomas is often contradictory, with most of the studies leaning towards the positive effect of immune effector cell infiltration on disease prognosis [54,55,56,57]. NK cell abundance in the tumor infiltrate positively correlates with increased overall survival in several sarcoma subtypes [58,59,60]. 

Additionally, early lymphocyte recovery after chemotherapy is associated with a better outcome in pediatric OS [61,62]. Combining surgery and polychemotherapy with systemic IL-2 treatment increases NK cell number and activity, with the magnitude of the increase correlating with an improved clinical outcome [63]. Finally, some studies have shown NK cells to be significant contributors to the control of sarcoma metastases [64,65,66,67,68].

**Figure 1:**
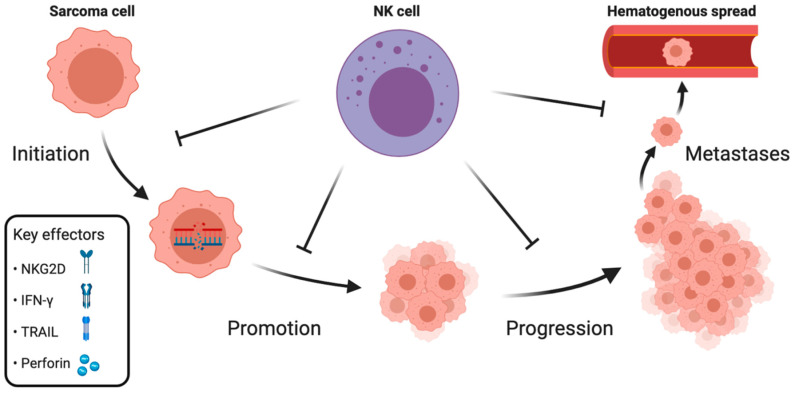
Overview of the central role of natural killer (NK) cells in all stages of sarcoma evolution. Through cell-mediated cytotoxicity, NK cells are able to inhibit tumor initiation, promotion, progression, and development of metastases. The key molecules necessary for NK cell-mediated tumor immunosurveillance are NKG2D, interferon (IFN)-γ, TNF-related apoptosis-inducing ligand (TRAIL), and perforin. Created with BioRender.com.

The aforementioned clinical data implicate the role of NK cells in controlling sarcomas′ growth. To summarize the available knowledge on NK cell-mediated immunosurveillance of sarcomas, we divide the evidence into three sections, categorizing NK cell’s role in: initiation and promotion, progression, and metastases (Figure 1). 

### 2.1. Initiation and Promotion

Tumor initiation is the first step of cancer development during which, by rising mutational load, healthy cells transform into cancer cells. It can be followed by tumor promotion, where transformed cells undergo clonal proliferation and form a tumor. During these early stages, it is up to the immune system to eradicate the newly developed neoplastic cells before progression and dissemination occur. 

A common carcinogenesis model is based on methylcholanthrene (MCA), which induces chemical mutagenesis and fibrosarcoma development upon inoculation. Smyth et al. evaluated fibrosarcoma formation in mice deficient in NK, Natural Killer T (NKT) cells, or both [69]. Both NK cells and NKT cells seem to be essential for host protection against MCA-induced sarcoma [69]. NK protective function against sarcoma could be enhanced by IL-12 therapy [69]. Later, the group has confirmed the crucial role of NK cells in preventing the formation of MCA-induced sarcoma and studied the pathways responsible for the recognition of transformed cells. Antibody-mediated neutralization of NK cell-activating receptor NKG2D increased mice susceptibility to MCA-induced sarcoma formation. The importance of the NKG2D was additionally underlined in IFN-γ^−/−^ and TRAIL^−/−^ mice, whereas mice depleted of NK cells, T cells, or deficient for perforin did not display any NKG2D-dependent changes in susceptibility. IL-12 therapy augmenting NK cell function and suppressing MCA-induced sarcoma formation was also dependent on the NKG2D pathway. Although NKG2D ligand expression is variable and often not detectable on sarcomas originating in wild type (WT) mice, sarcomas derived from perforin-deficient mice were RAE-1^+^ and immunogenic when transferred into WT syngeneic mice. These findings suggest an essential role of the NKG2D-perforin axis in control and shaping the early events of tumor formation [70]. On the other hand, another NK cell receptor, NKp46, is not associated with the surveillance of MCA-induced fibrosarcoma. However, tumors originating in NKp46^−/−^ mice implanted in WT mice induce a potent immune response suggesting a role of NKp46 in tumor immunoediting [71]. 

NRLP3 inflammasome, a cellular structure crucial for inducing and sustaining immune response, promotes tumorigenesis in specific cancer types. The deletion of NRLP3 has a protective effect in the MCA-induced fibrosarcoma model, dependent on NK cells and IFN-γ [72]. NRLP3 activation was also shown to impede NK cell antimetastatic function by decreasing NK cell tumor homing [72]. The molecular mechanisms of pro-tumorigenic role of NRLP3 vary between cancer types and are discussed in-depth in [73]. 

Carcinogenesis is also driven by oncogenic viruses such as HBV, HPV, EBV, and HHV-8 (KSHV). The latter is known to induce Kaposi Sarcoma (KS), with acquired immunodeficiency syndrome (AIDS) immunocompromised individuals being especially prone. Sirianni et al. showed that cells latently infected with KSHV are efficiently lysed by NK cells from healthy individuals [74]. However, the study yields specific limitations as the target cells were carefully selected based on their susceptibility to NK cell cytotoxicity [74]. On the contrary, Matthews et al. reported average levels of classical MHC class I, ICAM-1, HLA-E, and NKG2D ligands on latently infected primary fibroblasts, which caused a limited activation of resting NK cells [75]. Interestingly, infected cells were efficiently cleared by IL-15-primed NK cells [75].

A large study including over 1100 patients investigated the association between the HLA-KIR polymorphism and KSHV and KS status, finding that, in patients with KIR3DS1 plus HLA-B Bw4-80I, the KSHV seroprevalence was 40% lower, but the KS risk was two-fold higher. Similarly, the KSHV seroprevalence was 40% lower, but the KS risk 80% higher with HLA-C group 1 homozygosity. These data suggest that KIR-mediated NK cell activation may decrease KSHV infection’s risk but enhance KS progression if infection occurs [76]. Peripheral blood (PB) NK cell counts, on the other hand, do not correlate with the risk of KS development [77].

### 2.2. Progression

Cancer initiation and promotion can be followed by progression if not controlled by the immune system. Progression is the last phase of localized tumor development, characterized by increased growth speed and acquiring invasive potential. 

Mice selectively depleted of NK1.1 positive cells demonstrated more rapid initial growth upon injecting MCA207 sarcoma cells. In addition, large (20 mm) implanted MCA207 sarcomas were rejected following cyclophosphamide and IL-12 treatment, but the time to tumor eradication was significantly longer in mice with depleted NK cells [78]. Other groups confirmed that MCA-induced sarcoma growth could be reduced by IL-12 treatment, with the effect being mediated by NK cells [69]. Takeda et al. provided evidence of NK cells playing a key role in limiting the L929 TRAIL-sensitive fibrosarcoma progression in a subcutaneous murine model. The effect was mediated in a TRAIL and IFN-γ dependent fashion. The TRAIL pathway’s protective effect was dependent on NK cells and IFN-γ, supported by gene knockout experiments [79]. 

One of the most critical NK cell functions is to enhance the infiltration of other immune cells into the TME. In MCA-induced sarcomas, NK cells are known to infiltrate the developing tumors in early stages, with the semi-mature CD27^high^ NK cells being the predominant subpopulation of NK cells accumulating in the TME. The tumor-infiltrating NK cells display an activated surface phenotype and provide an early source of IFN-γ attracting other immune cells. Interestingly, host IFN-γ is critical for NK cell tumor homing, and, conversely, the tumor-infiltrating NK cells mainly suppressed tumor growth via the IFN-γ pathway. This implicates the importance of IFN-γ as a positive regulatory factor for both NK cell recruitment into the TME and an effective NK antitumor immune response [80]. NK cell-derived IFN-γ can also improve cancer cell recognition and associated NK cell cytotoxicity through ICAM-1 upregulation on cancer cells [81].

Moreover, IFN-γ plays a role in NK cell-mediated sarcoma immunoediting. Tumor cells isolated from immunocompetent mice displayed reduced expression of NKG2D ligand H60 and increased MHC class I expression compared with tumor cells isolated from mice treated with IFN-γ-specific neutralizing monoclonal antibody (mAb) [82]. IFN-γ can also induce programmed cell death ligand 1 (PD-L1) expression on cancer cells, subsequently inhibiting NK cell effector function [83].

Another approach to understanding the drivers of MCA-induced sarcoma progression was taken by O′ Sullivan et al. by comparing the gene expression between unedited and immunoedited tumors [84]. One of the most differentially expressed genes was *IL17D* encoding interleukin 17D (IL-17D), with a significantly increased expression in unedited tumor cells. Overexpression of IL-17D in edited tumor cells induced tumor rejection by stimulating CCL2 production from tumor endothelial cells, leading to an increase in the recruitment of NK cells. IL-17D-induced recruitment attracted mostly CD27^high^ NK cells, a semi-mature population of NK cells participating in IFN-γ-dependent T cell priming and contributing to suppressing tumor progression [84]. These data suggest that NK cells play a role in tumor immunoediting and suppressing sarcoma growth, both directly and indirectly by regulating other immune cells’ activity and infiltration. 

### 2.3. Metastases

The metastatic spread of neoplastic cells to distant anatomical regions is a leading cause of death in cancer patients. Metastatic spread is orchestrated by the intrinsic properties of cancer cells, enabling invasion of the local microenvironment and colonization of distant sites through lymphatic or hematogenous spread. Moreover, metastasis is regulated by microenvironmental and systemic processes, such as immunosurveillance.

NK cells are known for their antimetastatic potential [85,86,87,88]. Indicators of NK cell function such as high expression of NK cell-activating receptors and high cytotoxic or IFN-γ secreting properties have been linked to decreased metastatic load in multiple cohorts of cancer patients with risk of metastatic disease, suggesting their clinically relevant protective role [87]. High numbers of tumor-infiltrating NK cells have been inversely correlated with the presence of distant metastases in gastrointestinal stromal tumors (GIST), a subtype of sarcomas [64]. Interestingly, the incompatibility of nude mice as hosts for metastatic studies is attributed mainly to their NK cells, which efficiently remove circulating tumor cells [65]. NK cell-protective role against metastases was also recognized in multiple murine sarcoma models, where antibody- or cyclophosphamide-mediated NK cell depletion significantly increased metastatic load [66,67,68]. Studying the interactions between MHC class I expression, NK cells, and sarcoma metastases provided evidence for the correlation of RCT sarcoma metastatic potential with increased MHC class I expression, which in turn correlated with cancer cell resistance to NK cell lysis [67]. However, others did not observe any simple associations among MHC expression, development of metastases, and NK cells [89].

TNF-α, a highly pro-inflammatory cytokine secreted by effector immune cells, is one of the cytotoxic effector proteins capable of inducing cancer cell apoptosis. Surprisingly, in sarcomas, it was shown to have an NK cell-dependent prometastatic effect, indicated by selective antibody depletion experiments [86]. TNF-α can also exhibit prometastatic activity on its own through increased production of chemokines inducing angiogenesis and enhancing cancer cell motility, which has been thoroughly reviewed in previous publications [90,91]. Moreover, NRLP3 and IL-1R8 deficiencies were shown to have an antimetastatic effect attributed to enhanced NK cell function [72,92].

NK cells can be successfully used in metastases treatment; K562-expanded NK cells effectively eradicate Ewing sarcoma (EWS) metastases with little effect on the primary tumor in a murine model [93]. Furthermore, allogeneic hematopoietic stem cell transplantation (HSCT) was shown to inhibit the development of sarcoma metastases in an NK cell-dependent manner in clinical trials [94,95].

## 3. NK Cell Dysfunction in Sarcomas 

**Figure 2:**
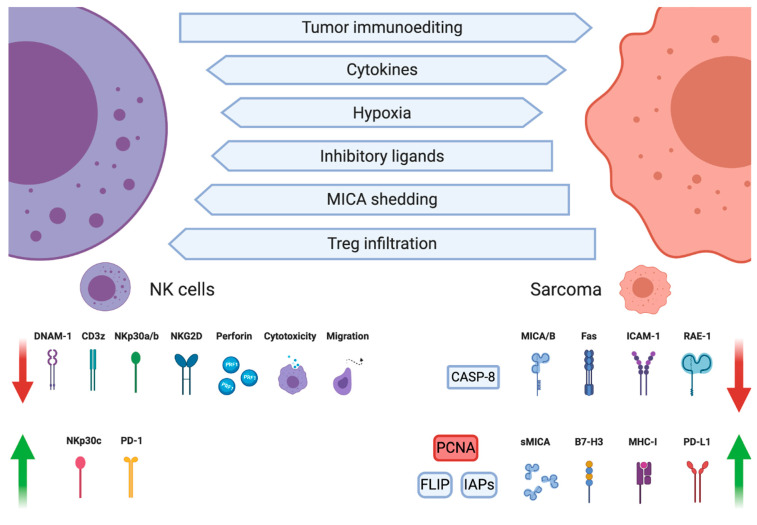
Interactions between natural killer (NK) cells, cancer cells, and tumor microenvironment (TME) shape sarcomas’ immunoevasion mechanisms. Tumor immunoediting, cytokines, hypoxia, and cells infiltrating the TME can change the sarcomas cell phenotype into an NK cell-resistant one, characterized by decreased expression of NK cell-activating ligands MHC class I-related chain A/B (MICA/B), retinoic acid early transcript 1 (RAE-1), intercellular adhesion molecule 1 (ICAM-1), and proteins necessary for Fas ligand (FasL)-mediated apoptosis (Fas, caspase-8). Conversely, expression of inhibitory molecules such as major histocompatibility complex (MHC) class I, programmed cell death ligand 1 (PD-L1), B7-H3 (CD276), proliferating cell nuclear antigen (PCNA), and antiapoptotic proteins cellular FLICE-inhibitory protein (c-FLIP), as well as inhibitors of apoptosis (IAPs), is increased. NK cell phenotype and function are also altered in the sarcoma TME by cytokines, hypoxia, and inhibitory ligands, resulting in a disturbed balance between activating and inhibitory receptor expression and associated cytotoxicity impairment. Created with BioRender.com.

In the course of cancer microevolution, neoplastic cells undergo a series of metabolic adjustments adapting the cells to increased proliferation. Unfortunately, the shift in cancers metabolic state is accompanied by creating a hostile TME inhibiting the anticancer immune response and promoting homing of immunosuppressive cells such as regulatory T cells (Tregs) or M2 macrophages (Figure 2). Already in 1981, Gerson et al. found that sarcomas substantially inhibit NK cell functions. NK proliferation in response to concanavalin A, macrophage migration inhibitory factor secretion, and cell-mediated cytotoxicity were all suppressed by macrophages infiltrating the sarcoma, with the cytotoxicity being the most preserved function [96]. 

Cytotoxicity impairment of PB NK cells was reported in chemotherapy-naïve sarcoma patients, in contrast to NK cells from renal cell carcinoma patients, which displayed normal cytolytic activity [97,98]. The cytotoxic function of NK cells could be restored by IL-2 and Hsp-70-derived TKD peptide. Additionally, NK cells in chemotherapy-naïve sarcoma patients had reduced proportions of mature CD56^dim^ population and slightly increased NKG2D expression compared to age-matched controls. After disease progression or relapse, NK cell phenotypic alterations were more remarkable; progressively reduced CD56^dim^ proportions and decreased expression of NKG2D, CD3ζ, perforin, together with reduced frequencies of differentiated CD57^+^ NK cells were all observed [98]. 

Suppression of the NK cell compartment increases at the tumor site. A significant decrease in the NK cell proportions is observed in tumor-infiltrating lymphocytes (TILs) compared to matched peripheral blood mononuclear cells (PBMCs). In contrast, no difference was observed between tumor-infiltrating and PB CD3^+^ bulk T cells, CD4^+^, and CD8^+^ T cells, indicating an impairment in NK cell tumor homing or intratumoral persistence [99]. Profiling of TIL NK cells provided evidence for decreased CD16, KIR2DL1, KIR2DL2/L3, and KIR3DL1 expression in the CD16^+^ KIR^+^ and CD16^+^ KIR^−^ NK cell subsets, compared to NK cells in matched PBMCs. DNAM-1 and NKG2D expression on TIL NK cells was also reduced in the vast majority of the patients compared to matched PBMCs [99]. Significantly, NKG2D and DNAM-1 downregulation might contribute to disease progression in these patients as sarcoma cells are mostly recognized by NKG2D and DNAM-1 receptors [43,97]. In GIST, the NKp30 receptor was preferentially downregulated on tumor-infiltrating NK cells. Interestingly, PB NK cells in GIST patients expressed immunosuppressive NKp30c isoform more frequently with proportionally less NKp30a and -b. The expression of NKp30c isoform was associated with an unfavorable clinical outcome [64].

The co-culture experiments of NK and primary sarcoma cells provided further insights into sarcoma-induced functional impairment. Sarcoma cells caused a decreased expression of NKG2D, DNAM-1, and interfered with IL-15-induced expression of NKG2D, DNAM-1, and NKp30, consecutively inhibiting the cytolytic activity of NK cells. The inhibition was contact-dependent, and the cytotoxicity impairment was directly linked to the downregulation of the respective NK cell-activating receptors. Five days of IL-15 pretreatment was able to increase NK cell resistance to sarcoma suppression. In opposite to the above-mentioned changes in TIL NK cells, CD16 expression and ADCC were not affected by the NK–sarcoma co-cultures [100]. 

Few reports show no differences in IFN-α signaling, NKG2D expression, and NK cytotoxic properties between PB NK cells from sarcoma patients and healthy donors [43,101]. However, the analyzed patient group was limited to freshly diagnosed patients with a most likely early-stage disease, which indeed can be associated with mild or no functional impairment in PB NK cells [86,98].

### 3.1. Tumor-Infiltrating Immunosuppressive Cells

Other cells in the sarcoma microenvironment can contribute to creating a suppressive milieu. Tumor stromal cells derived from fresh sarcoma samples display a potent antiproliferative effect on PBMCs, decrease NK cell cytotoxicity and NKp44/46 expression, as demonstrated in co-culture experiments [102]. Through rewiring chemokine and metabolic networks, sarcomas can induce immunosuppressive CD4^+^CD25^+^ Treg infiltration. Ghiringhelli et al. reported an inverse correlation between NK cell activation and Treg abundance in GIST patients [103]. By expressing membrane-bound transforming growth factor β (TGF-β), Tregs directly inhibit NK cell cytotoxicity, proliferation, and alter NK cell phenotype by downregulating NKG2D receptor expression [103].

### 3.2. Cytokine-Dependent Inhibition

Cytokines present in the TME can also suppress NK cell function. Hafner et al. showed that TNF-α suppresses NK cell cytotoxicity, consequently impairing NK cell antimetastatic function in murine sarcoma model [86]. The molecular mechanism of TNF-α-mediated NK cell suppression has not been fully elucidated, but new evidence indicates that TNF-α may contribute to NK cell exhaustion in a TIM-3-dependent manner [104]. The group has also provided insights into time-dependent changes in NK cell cytotoxic function after sarcoma tumor inoculation. At first, the NK cell activity increases, but after quickly reaching its peak it starts to decrease below the initial level [86]. On the other hand, TNF-α was shown to increase human OS cells′ susceptibility to NK lysis by CD54 and CD58 upregulation, demonstrating a dual role of TNF-α in NK-sarcoma interactions [105,106]. Notably, CD54 (ICAM-1) expression on cancer cells is essential for NK cell cytotoxicity, and its magnitude directly correlates with OS susceptibility to NK cell lysis [106,107,108].

Interferons are proinflammatory peptides known for their antiviral properties. One of their mechanisms of action is MHC class I upregulation, aiming to increase the presentation of viral peptides. In murine MCA sarcoma, IFN-γ and IFN-α were shown to reduce expression of NKG2D ligand H60. Downregulation occurred at the transcript level and was STAT1-dependent. IFN-γ-treated MCA sarcomas with initially high levels of H60 were resistant to killing by IL-2-activated NK cells. Resistance was not solely dependent on H60 downregulation but also on IFN-enhanced MHC class I expression [82].

TGF-β is another vital player in sarcoma TME. It regulates the extracellular matrix (ECM) protein composition and induces osteopontin synthesis in OS cells, increasing their malignant potential [109,110]. Additionally, Treg-derived TGF-β can inhibit NK cell effector functions such as cytotoxicity and tumor homing [111,112,113]. Besides, Gao et al. provided evidence for TGF-β-induced transformation of NK cells into intermediate type 1 innate lymphoid cells (intILC1) and ILC1 in the sarcoma microenvironment. Importantly, intILC1s and ILC1s did not provide sufficient control of local tumor growth and metastasis, whereas NK cells favored tumor immunosurveillance. ILC1-derived TNF-α was suggested to be partially responsible for an escape from the innate immune system [114]. In soft tissue sarcoma patients, high TGF-β1 intratumoral expression is associated with aggressive disease and shorter disease-specific survival [115]. 

### 3.3. MHC-Dependent Inhibition

MHC class I molecules serve as ligands for inhibitory KIR and NKG2A receptors. Current evidence shows that chemotherapy can increase classical and nonclassical MHC class I molecule expression in OS cells, consequently inhibiting NK cell activity [43]. One of the critical NK-suppressive MHC molecules is peptide-loaded HLA-E, which can be expressed in different tumor cell types, including sarcomas. It is a potent inhibitor of NK cell activity, acting via NKG2A [116]. Moreover, it has been shown that EWS treated with anti-GD2 chimeric antigen receptor (CAR)-NK cells developed resistance to the treatment in an HLA-G-dependent manner, which was selectively upregulated on tumor cells only in CAR-treated mice. NKG2A knockdown restored CAR-NK lytic function and allowed for effective tumor eradication [117]. In OS patients, the MHC class I expression itself is associated with a better prognosis, most likely due to T cell-mediated immune response [118].

Interestingly, the relationship between NK cell activation and MHC I expression appears to be nonlinear. A moderate increase of MHC class I expression on EWS cells caused a highly NK-resistant phenotype, whereas downregulation of MHC expression did not change the susceptibility, implicating the existence of a threshold. That, in turn, would allow modest changes in the target cell surface phenotype to significantly affect the susceptibility to NK cell-mediated lysis [119]. Not only the surface expression but also the KIR-HLA mismatch degree between NK cells and OS determines their susceptibility to NK cell lysis [120].

### 3.4. MICA Shedding

Shedding of NK cell-activating ligands can also contribute to sarcoma immunoevasion. NKG2D ligand MICA is shed in a matrix metallopeptidase (MMP)-9-dependent manner in OS. Soluble MICA (sMICA) was shown to cause NKG2D downregulation, impairing NK cell response [121]. High concentrations of sMICA were correlated with poor prognosis in multiple cancer types [122]. In sarcomas, sMICA concentration is increased in advanced disease, downmodulating NKG2D expression on NK cells. On the contrary, most of the early stage and well-differentiated sarcomas were shown to express MICA on the cancer cell surface, indicating that MICA expression is lost along the disease progression [41]. Therefore, MICA shedding might be an early event in sarcoma immunoevasion, contributing to the disease progression [42,123]. Multiple studies have shown that increased MICA and sMICA expression were associated with decreased NKG2D expression on NK cells and correlated with advanced and metastatic disease [41,42]. Further, MMP-9 and MMP-2 expression is associated with the presence of metastasis and poor survival in OS patients and could be potentially used as a prognostic biomarker [124,125]. 

### 3.5. Apoptosis Resistance

Along with the tumor microevolution, neoplastic cells can acquire resistance to cell-mediated cytotoxicity. Resistance to FasL-mediated apoptosis can be mediated through downregulated caspase-8 and increased expression of antiapoptotic proteins such as cellular FLICE-inhibitory protein (c-FLIP) or Inhibitors of apoptosis (IAPs). Such mechanism has been demonstrated to play a role in immunoevasion of rhabdomyosarcoma (RMS) and EWS cell lines, as well as primary EWS samples [46,47]. Notably, FasL is constitutively expressed in the lung, implicating that Fas expressing cancer cells should be eliminated by lung endothelium. However, metastatic OS acquire resistance to Fas-mediated death by Fas downregulation, allowing for lung colonization [126]. Chemotherapeutic agents such as gemcitabine upregulate Fas surface expression and may therefore be an important part of multimodal therapy for OS lung metastases [127]. Cisplatin treatment can overcome FasL resistance by downregulating c-FLIP-L, sensitizing OS cells to FasL mediated apoptosis [128]. Sensitization of OS cells to FasL can also be induced by histone deacetylase inhibitor (HDACi) entinostat, which increases Fas transcription, its localization in membrane lipid rafts and decreases the expression of antiapoptotic c-FLIP [129,130].

### 3.6. Immune Checkpoints

Immune checkpoints are molecules regulating the activity of immune system, which in physiological setting play a protective role against autoimmunity and overactivation of lymphocytes. Programmed cell death protein 1 (PD-1) is a well-known checkpoint molecule, functioning as a “break” in the immune system [131]. Typically, only a small fraction of PB NK cells express PD-1, but the proportion is increased on NK cells in cancer patients [132]. PB NK cells from KS patients exhibit PD-1 expression in a CD56^dim^CD16^+^ population with otherwise normal surface phenotype. However, despite the normal phenotype, PD-1^+^ NK cells demonstrated reduced cytotoxicity and IFN-γ production ex vivo following the direct triggering of NKp30, NKp46, CD16, or short stimulation with target cells, suggesting a role of PD-1 in KS-mediated NK cell exhaustion [133]. Moreover, PD-L1 expression in OS cell lines determined their susceptibility to NK cell lysis, shown by mAb blocking experiments [134]. However, in OS patients, intratumoral PD-L1 expression positively correlated with increased immune infiltration including NK cells and, surprisingly, event-free-survival [57].

B7 homolog 3 (B7-H3, CD276) is an immune checkpoint protein of the B7-CD28 family with a vital role in T cell inhibition [135]. B7-H3 overexpression is observed in multiple cancers, including OS, RMS, and EWS [135,136,137,138]. B7-H3 is expressed in 91.8% of OS tissues, where it promotes OS cell invasion and is inversely correlated with TIL abundance [136]. High B7-H3 expression is also associated with shorter survival and disease recurrence [136]. 

Interleukin-1 receptor 8 (IL-1R8) is a member of the IL-1 receptor family, acting as a negative regulator of other IL-1 receptor and Toll-like receptor (TLR) signaling, that has been recently established as a checkpoint molecule in NK cells. Knockout of IL-1R8 has been shown to restore NK cell antitumor function in MCA-induced sarcomas, implicating a role of IL-1R8 in sarcoma-mediated NK cell suppression [92]. Expression of other checkpoint molecules such as T-cell immunoglobulin and mucin domain-3 (TIM-3) ligands and NKp44-inhibiting proliferating cell nuclear antigen (PCNA) was also reported in sarcomas [45,139]. 

### 3.7. Altered Oxygen Metabolism

Rapid cell turnover and cancer growth are associated with decreased O_2_ gradient as tumors grow beyond their vascular supply. Large murine sarcomas contain a severely hypoxic core, whereas smaller tumors possess hypoxic gradients throughout the tumor mass. Evidence indicates that these hypoxic gradients orchestrate sarcoma cell migration and ECM remodeling, increasing their metastatic potential. Additionally, hypoxia-inducible factor 1-alpha (HIF-1α) increases CXCR4 expression on sarcoma cells, contributing to metastasis development. Notably, in sarcoma patients, increased HIF-1α and CXCR4 expression are associated with advanced disease [140,141].

A hypoxic microenvironment is reported to alter the susceptibility of human OS cells to NK cell-mediated lysis. Different OS cell lines expressed various NKG2D ligands such as MICA, MICB, and ULBP1/2/3, with the MICA being most frequently expressed [44]. In a HIF-1α mediated way, hypoxia decreased cell surface MICA expression without increasing the secretion of soluble MICA, resulting in reduced susceptibility of the OS cells to the NK cell-mediated lysis [44]. Moreover, by inducing HIF-1α, hypoxia impairs NK cell function by inhibiting their response to activating cytokines as well as suppressing cell-mediated cytotoxicity capabilities, except for ADCC [142]. Significantly, in STS tumors, low oxygen content is associated with poor disease-specific and overall survival [143]. Additionally, bone and soft-tissue sarcomas are characterized by increased oxidative stress, which is known to inhibit NK cell effector functions [144,145].

### 3.8. HIV-KS-NK Cell Axis

NK cell-mediated immunity is significantly impaired in AIDS patients with progressing KS compared to both HIV-negative patients with indolent classic KS and healthy blood donors. The highly active antiretroviral therapy (HAART) is able to rescue impaired NK cell function in AIDS-KS patients, inducing tumor regression and HHV8 clearance. However, AIDS-KS patients with more aggressive disease and no response to therapy had persistent HHV8 viremia and reduced NK cell cytotoxicity. These results suggest a crucial role of NK cells in the control of HHV8 infection and KS tumor, as well as AIDS role in mediating NK cell suppression [74]. Additionally, NKG2C^+^ NKp46^low^ NK cells were discovered to form a novel, poorly functional subset present in AIDS-KS patients [146]. 

HIV-negative classical KS patients have significantly decreased NK cell cytotoxicity compared to healthy controls, whereas healthy HHV8 carriers have phenotypically impaired NK cells with reduced expression of NKp30, NKp46, and CD161 receptors [147]. Further, KS patients show downmodulation of NKG2D, associated with impaired NK-cell lytic capacity, which could be restored upon KS treatment [148]. Interestingly, KS cells exhibited high expression of NKG2D ligands confirmed in situ by immunohistochemical (IHC) staining of KS biopsies. However, no tumor-infiltrating NK cells were detected, suggesting a defect in NK cell homing or persistence in the KS microenvironment [148]. PGE_2_ was identified as a critical inhibitory mediator responsible for impairing NK cell response in KS, acting by down-modulation of NKG2D expression on resting NK cells and impairing IL-15 induced proliferation and phenotypic changes [148]. Other studies have demonstrated that PGE_2_ inhibits NK cell migration properties and impairs their accumulation in TME, which could be the reason for the lack of NK cells among the KS TILs [149,150]. Moreover, KSHV proteins K3 and K5 were shown to drastically downregulate ICAM-1, MICA, MICB, and AICL (NKp80 ligand) expression on infected cells, increasing their resistance to NK cells [151,152,153].

### 3.9. Iatrogenic NK Cell Suppression

NK cell functional impairment can be iatrogenic. Surgery was shown to transiently impair NK cell cytotoxicity; however, the phenomenon’s significance is unclear [154]. While in other cancers chemotherapy is reported to upregulate NK cell-activating ligands, a comparison of pre- and post-chemotherapy OS tissue sections provided evidence for either unaltered or decreased expression of MICA, CD112, and CD155 after chemotherapy [43]. Moreover, depending on the specific agent and the dose, chemotherapy can also directly suppress NK cell function [154,155,156,157]. Zoledronic acid (ZA), tested as maintenance therapy in clinical trials in patients with bone sarcomas (OS and EWS), acts by inhibiting bone resorption and inducing apoptosis in osteoclasts and tumor cells. Its effect on NK cell activity is, however, unfavorable as ZA can impede in vitro NK cell expansion and cytolytic responses to EWS, raising concerns against combining NK cell therapies with ZA in bone sarcoma treatment [158].

## 4. NK Cell-Based Therapies in Sarcomas

Numerous immunotherapeutic approaches have been tested in patients with sarcomas, and the results have not been as impressive as in the treatment of other solid tumors. In theory, proper management of immune checkpoint signaling by monoclonal antibodies can be one of the modalities to revive the functioning of NK cells within the tumor [159]. Immune checkpoint blockade therapy with antibodies blocking target cytotoxic T lymphocyte-associated antigen 4 (CTLA-4) and the PD-1/PD-L1 pathway leads to durable clinical responses in an increasing number of solid tumors. Unfortunately, responses in patients with sarcomas have been observed infrequently, except for the TIL-rich undifferentiated pleomorphic sarcoma [160,161,162]. Preliminary clinical evaluation of PD-1-PD-L1 axis inhibition in angiosarcoma and alveolar soft part sarcoma also shows promising results [163,164,165]. 

**Figure 3:**
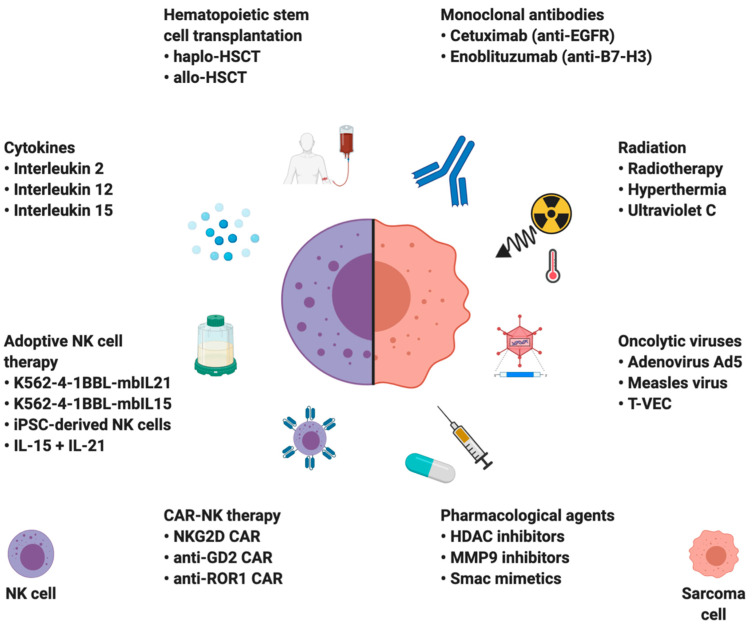
A schematic illustration of selected prospective sarcoma treatment modalities based on: modifying natural killer (NK) cell properties (left); or sensitizing sarcoma cells to NK cell cytotoxicity (right). Created with BioRender.com.

One of the reasons for the inefficacy of immune checkpoint inhibition therapy is the fact that sarcomas have fewer TILs per gram of tissue and lower ratios of TIL infiltration when compared to other cancers, e.g., melanoma or renal cell carcinoma [53]. However, primary sarcomas and sarcoma cell lines were shown to be one of the most vulnerable tumor types to spontaneous NK cell cytotoxicity, making NK cell-based therapies novel and promising treatment alternative [30,31,32]. The NK cell-based immunotherapies can be divided into two groups based on their principle of action: strategies augmenting NK cell function and those sensitizing cancer cells to NK cell-mediated lysis (Figure 3).

### 4.1. Hematopoietic Stem Cell Transplantation (HSCT)

HLA-mismatched HSCT combines the effects of chemotherapy and graft-versus-tumor (GvT) phenomenon. HSCT with grafts from haploidentical donors was shown to be safe and beneficial in pediatric solid tumors including sarcomas [166,167]. Already in 1984, a clinical trial in Moscow demonstrated that allogeneic bone marrow transplant suppressed lung metastases development in a group of OS patients after radical surgery. Importantly, all of the treated patients who did not develop metastases had normal NK cytotoxicity levels, whereas, in the metastases group, the NK activity was significantly lower, suggesting a critical role of NK cells in suppressing sarcoma metastases [94]. A clinical trial in pediatric cancer patients, including sarcomas, has shown that HSCT’s effects could only be observed in patients with mismatched KIR-HLA [168]. However, the beneficial effect of HLA-mismatched grafts was associated with a higher risk of toxicities [169]. A potential role for NK cells in the graft-versus-tumor effect is supported by in vitro studies showing that the KIR-HLA mismatch degree can predict OS cell lines susceptibility to NK cell lysis [120]. Another study retrospectively investigated allo-HSCT in RMS patients and reported moderate results, prompting further investigations and suggesting the potential use of allo-HSCT as a consolidation therapy [169]. A case report study has also shown haplo-HSCT to be effective in metastases control in two patients with stage IV EWS [95]. Further clinical trials are being carried out to determine the clinical utility of HSCT in high-risk sarcoma patients; however, there are currently no recommendations to use HSCT as a sarcoma therapy irrespectively of the disease stage.

### 4.2. Cytokines

Because of their potent immunostimulatory properties, cytokines have always raised interest as adjuvant therapies. The antitumor activity of the IFN-α-conjugated antibody against the OS cell line was first reported in 1984 by Flannery et al. The treatment resulted in a modest increase in NK cell cytotoxicity, attributed to IFN-α induced activation [170]. Multiple studies have shown the efficacy of IFN-α, IL-2, IL-12, IL-15, IL-18, and IL-28 in augmenting NK cell functions by increasing their cytotoxicity, rendering resistant to TME-mediated suppression, and altering sarcomas adhesion molecule profile. Such an approach was proven useful through in vivo studies in multiple sarcoma subtypes [43,78,97,171,172,173,174,175,176,177,178,179]. Because of synergistic effects between cytokines and chemotherapy, they can be used together in sarcoma treatment. A combination of chemotherapy and NK cell-activating cytokines has been shown to induce regression of both, sarcoma primary tumors and lung metastases in murine models [78,175,176]. Importantly, the cytotoxic effect against EWS and RMS cells is almost entirely dependent on NKG2D and DNAM-1 receptors in resting NK cells. However, IL-15-treatment decreases the dependency on NKG2D and DNAM-1, reducing the probability of immune evasion by downregulating their respective ligands [97,174]. 

Pegylated IFN-α-2b was tested as maintenance therapy in OS in phase III clinical trial NCT00134030. The results show no benefit of IFN-α therapy and poor treatment tolerance due to associated toxicities [180]. IL-2 treatment was also tested in pediatric OS patients [63]. Unfortunately, systemic cytokine therapies are associated with severe side effects [181,182]. An exciting alternative is direct intranasal therapy with either adenoviral or polyethyleneimine vector encoding IL-12, which can locally increase NK cell antitumor potential and lead to the eradication of OS lung metastases [182,183,184]. 

A different way of localized cytokine treatment is based on isolated limb perfusion. After sarcoma surgery, blood vessels of the treated limb are being reconnected to form a closed system with a pump and treated with extremely high concentrations of melphalan, TNF-α, and mild hyperthermia [185]. Such an approach has proven to be useful by allowing a higher percentage of limb-sparing surgeries and achieving greater response rates [185].

### 4.3. Monoclonal Antibodies

Sarcomas can also be targeted by monoclonal antibodies (mAb). Advantages of mAb therapy include the preservation of NK cell ADCC capabilities under hostile sarcoma TME conditions as well as the presence of a synergy between mAb treatment and chemotherapy, which increases ADCC-sensitivity of sarcoma cells [100]. Other agents, such as lenalidomide, can also enhance ADCC against sarcomas [186]. 

Epidermal growth factor receptor (EGFR) is commonly expressed on OS cell lines and 90% of primary OS samples, with high expression correlated to large tumor volume, prompting to utilize the anti-EGFR monoclonal antibody cetuximab as a potential therapy [187,188]. Unfortunately, no responses to cetuximab were observed in phase II clinical trial in sarcomas indicating that no further trials should follow unless new predictive markers were discovered [189].

Enoblituzumab (MGA271), a humanized IgG1 monoclonal antagonistic B7-H3 antibody, has been studied in phase I clinical trial in patients with refractory B7-H3-expressing neoplasms such as melanoma and advanced solid tumors (NCT01391143). Most of the patients experienced stable disease and significant tumor shrinkage with no dose-limiting toxicity and good tolerance. Another ongoing clinical trial is investigating enoblituzumab in B7-H3-expressing pediatric solid tumors, including neuroblastoma, RMS, OS, EWS, Wilms’ tumor, and desmoplastic small round cell tumors (NCT02982941) [190]. A different mAb-based approach is based on insulin-like growth factor receptor (IGFR)-1 antibody-mediated inhibition, which has shown a single-agent efficacy in a subset of EWS patients [191]. Interestingly, IGFR-1 inhibition enhances NK cell expansion without impairing the NK cell-mediated lysis of EWS cells [192]. 

### 4.4. Immunomodulation

As discussed above, through NK cell-mediated immunoediting, selective pressure favors persistence of NK cell-resistant sarcoma cells expressing low levels of NKG2D and DNAM-1 ligands and high levels of MHC class I. Increasing the expression of NK cell-activating molecules on cancer cells, thus their sensitivity to NK cell-mediated lysis could serve as an important part of multimodal therapy in future sarcoma treatment. Sodium valproate (VPA), a histone deacetylase inhibitor, increased surface MICA and MICB expression in multiple OS cell lines and, consequently, their susceptibility to NK cell lysis. By inducing acetylation of histones bound to *MICA* and *MICB* gene promoters, VPA increases only the cell-surface but not soluble MICA and B expression [193]. Another study by the same group confirmed this finding and found that VPA synergizes with hydralazine in sensitizing OS cells to NK lysis. The co-treatment additionally upregulated Fas expression, increasing OS cells susceptibility to FasL mediated death [194]. 

A narrow-spectrum HDACi entinostat has been shown to increase both NKG2D expression on NK cells and MICA/B in multiple tumor target cells, including sarcomas, augmenting NK cell cytotoxicity in vitro and in vivo and consequently suppressing sarcoma lung metastases in mice [195]. Further, entinostat downregulates c-FLIP expression concomitantly redirecting Fas cellular localization to the lipid rafts, sensitizing sarcoma cells to FasL-mediated apoptosis [129,196]. Other groups have shown that entinostat increases the expression of MICA/B, ULBP1/2/5/6, and CD155 in OS, RMS, and EWS cells, enhancing NK cell cytotoxicity [197,198]. However, in a nude mice OS lung metastases model, no significant effects of the treatment were observed. The lack of efficacy was linked with the failure of NK cells to penetrate inside the tumor nodules [197]. Besides, although preliminary studies report entinostat-mediated enhancement of NK cell effector function, both HDAC inhibition and DNA hypomethylation have been linked to NK cell cytotoxicity impairment, raising caution against combining them with NK cell-based therapies [198,199,200]. 

NKG2D ligands are upregulated following genotoxic stress induced by, e.g., RT, through activation of ATM and ATR pathways, consecutively alerting the immune system about potentially dangerous transforming cells [201]. Combining radiation and NK cell treatment has shown promising results in preclinical studies targeting sarcomas and sarcoma stem cells. Radiation increases the expression of NK activating ligands MICA and MICB, as well as Fas, both in vivo and in post-RT patient biopsies. The effects were preferentially observed on cancer stem cells, increasing their sensitivity to NK lysis [202,203,204]. Another way to increase NKG2D ligand expression is blocking MMP-9-mediated MICA shedding. Using a specific MMP-9 inhibitor might represent a double-benefit therapy, where it can both inhibit tumor invasion and restore NK cell-mediated antitumor immune response [123].

A novel and promising means of cancer therapy are based on infecting tumor cells with modified oncolytic viruses such as adenovirus serotype 5, herpes simplex or measles virus. Such an approach has been shown to increase surface expression of NKG2D ligands in multiple sarcoma cell lines [205,206]. Additionally, viral proteins, e.g., Ad 2/5 E1A, can also be targeted by NK cells [207]. Virus-infected sarcoma cells were shown to induce increased secretion of perforin, granzyme B, IFN-γ, TNF-α, granulysin, and sFasL in NK cells [205]. Phase II clinical trial (NCT03069378) demonstrated antitumor activity of Talimogene Laherparepvec (T-VEC), a genetically engineered oncolytic herpes simplex virus, combined with anti-PD-L1 mAb pembrolizumab in locally advanced or metastatic sarcomas [208]. 

As discussed above, tumor cells can develop resistance to NK cell-induced apoptosis. Smac mimetics (SM) are small molecules that, by antagonizing IAP proteins, can compensate for their overexpression. In addition to chemotherapeutic agents, SM-treatment was proven to effectively sensitize sarcoma cell lines toward TRAIL-mediated NK cell killing [47,209]. Additionally, SM’s presence during IL-2 priming increases NK cell cytotoxic capacity via activation of the TNF-α–NF-kB axis, prompting to use SM as a novel strategy acting through cancer cell sensitization and NK cell functional enhancement [47].

Regional hyperthermia has shown a benefit in certain clinical trials in soft tissue sarcoma treatment as an addition to chemotherapy [210,211]. It has been shown that increased temperature induces HSP72 expression, consecutively increasing OS, EWS, and chondrosarcoma cells susceptibility to NK cell-mediated lysis [212,213]. Other promising immunomodulatory strategies relying on NK cells include ultraviolet c (UVC) irradiation [214], spironolactone [215], tilorone analogs [216], cytokines [217,218], lenalidomide [186], imatinib [219], and low-level γ irradiation [203], which were all proven effective in preclinical settings. 

### 4.5. Adoptive NK Cell Therapies

Adoptive cell-based immunotherapies are based on transferring auto- or allogenic cells capable of eliciting an effective anticancer immune response. The greatest advantage of adoptive therapies is the ability to combine intrinsic cell properties with function-enhancing gene edits and ex vivo cell activation. Because of the NK cell’s crucial role in the control of sarcomas, they are intensively investigated as novel means of treatment. Multiple studies have shown that the adoptively transferred NK cells are able to infiltrate sarcoma lung metastases much more effectively than the primary tumor site and mount a potent immune response against metastases, but not the primary tumor [93,94,95]. Since lung metastases are a primary cause of death among sarcoma patients, NK cell therapy combined with regional tumor management could be effectively used to treat the patients [36,220,221]. Unsatisfying results of HSCT in sarcoma treatment resulted in further attempts at improving the results by administrating IL-15 activated NK cells after haplo-HSCT for pediatric sarcoma treatment. The therapy was safe and most of the patients responded; however, the results were moderate and short-lasting [222].

Autologous or allogeneic NK cells can be expanded to large numbers using K562-based feeder cells expressing 4-1BB-L and membrane-bound (mb) IL-15 or mbIL-21 [223,224]. To generate a sufficient number of cells, the NK cells are expanded for approximately three weeks, depending on the protocol [225]. The doses of expanded NK cells, characterized by potent antitumor activity, range from 10^6^ to 10^7^ cells/kg [225]. Although there is evidence of in vivo proliferation of transferred cells, multiple infusions in 2–3-day intervals are often used to obtain maximal antitumor effect [225,226,227]. Additionally, the expansion process allows for genetic modifications of NK cells, which have always been problematic in primary cells [223,228]. Evaluation of expanded NK cells against ex vivo cancer cells isolated from primary pediatric tumors found EWS and RMS especially sensitive to expanded NK cells [30]. Based on these findings, a clinical trial of expanded haploidentical NK cells in the EWS and RMS patients has been started (NCT02409576). Another group has shown promising results in targeting OS with expanded NK cells [215]. A combination of expanded NK cells and RT has proven to be effective in a first-in-dog clinical trial, showing synergy between RT and expanded NK cells. RT increased sarcomas’ susceptibility to NK cell cytotoxicity and improved tumor homing of adoptively transferred cells, providing a rationale for testing such combination in clinical settings [229]. In mice, expanded NK cells have been shown to effectively eradicate EWS lung metastases with no significant effect on the primary tumor, urging for advancements in improving primary tumor homing of adoptive NK cells [93]. Metastatic pediatric solid tumors such as OS, neuroblastoma, and glioblastoma can be potentially targeted with expanded NK cells combined with IL-15 superagonist (N-803) and TIM-3 blockage [230]. Overexpression of DNAM-1 in expanded NK cells also yields promising results, due NK cell cytotoxicity’s enhancement proven on a dozen sarcoma cell lines [45]. Multimodal immunotherapy based on K562-4-1BBL-mbIL-15-expanded NK cells and an anti-CXCR4 mAb has shown robust antitumor activity in EWS and RMS models. The synergy between NK cell antimetastatic function and inhibition of prometastatic CXCR4 on sarcoma cells resulted in the elimination of primary tumors as well as micro- and macrometastases in mice [231]. Interestingly, NK cell expansion does not have to be limited to feeder cells. Feeder-free two-stage expanded NK cells combined with RT were shown effective in preclinical RMS models [204]. 

Unfortunately, adoptive therapies with expanded NK cells are not as safe as initially thought. A clinical trial of MHC-matched, T-cell depleted PB stem cell transplantation with donor-derived K562-4-1BBL-mbIL-15-expanded NK cell infusions in pediatric sarcomas (NCT01287104) reported acute GvHD in five of nine participants, attributed to expanded NK cells [232]. Nevertheless, the majority of adoptive NK cell clinical trials demonstrate a very low incidence of GvHD [233]. There is also evidence for the protective role of NK cells against GvHD, mediated through lysis of alloreactive T cells [234].

Chimeric antigen receptors (CARs) are an effective way of targeting tumor cells. Ganglioside GD2 is a commonly expressed antigen among young sarcoma patients. Its expression is stable and unaffected by recurrences, making an anti-GD2 therapy an excellent second-line treatment alternative [235,236]. A GD2 mAb blocking therapy was shown to inhibit tumor viability by itself and enhanced the proapoptotic effects of cisplatin in OS cells [237]. Expression of CARs directed against the GD2 in activated NK cells has proven to increase NK cells’ activity against EWS in an antigen-specific manner [117]. Surprisingly, the adoptive transfer of GD2-specific CAR-NK cells failed to eliminate GD2-expressing EWS xenografts due to CAR-NK-induced upregulation of inhibitory HLA-G1 on tumor cells [117]. HLA-E can also exhibit potent immunosuppressive effects on NKG2A^+^ NK cells, prompting to block NKG2A expression on expanded NK cells to enhance their antitumor properties [116]. Receptor tyrosine kinase-like orphan receptor 1 (ROR1) is another potential target tested in the treatment of solid tumors. It is widely expressed across different sarcoma subtypes, and in vitro studies have proven anti-ROR1 CAR NK cells to effectively induce U2OS cell lysis, accompanied by increased IFN-γ secretion [238,239]. Interestingly, a chimeric NKG2D receptor transduced T cells and NK cells were successfully used in preclinical models of EWS and OS [240,241,242].

Not only NK cells, but also NK cell line NK-92 is being tested as an immunotherapeutic tool in clinical trials in multiple cancer types. In a phase I clinical trial with sarcoma patients, NK-92 therapy was found safe but ineffective, possibly be due to IL-2 dependency of NK-92 cells and associated poor in vivo persistence [243]. On the other hand, a recently published preclinical study has shown that NKG2D and/or DNAM-1 overexpression in NK-92 cells robustly increased their cytotoxicity towards multiple sarcoma explants [99]. A case report of repeated NK-92 cell intratumoral injections combined with systemic chemotherapy in a relapsed multifocal EWS patient reported a moderate antitumor activity limited to the injection site. The authors attributed the effect to the NK-92 cells, as no response was observed in tumor sites where no cells were administered [244].

Sarcoma’s tendency to hematogenous spread and lung colonization poses a great therapeutic challenge. A combination of IL-2 in aerosol and NK cell infusion effectively eradicates lung metastases in a preclinical murine model. Delivery of IL-2 in aerosol selectively increased lung homing of transferred NK cells and lacked systemic toxicities [245]. Another strategy aiming at eradicating sarcoma lung metastases is based on CXCR2 overexpression in the NK-92 cell line, which increased their homing to OS lung metastases and improved therapeutic effect [246].

Finally, iPSC-derived NK cells (iPSC-NK) represent a promising therapeutic modality for the next-generation NK cell adoptive immunotherapy [247]. iPSCs offer a versatile platform to generate unlimited doses of homogenous NK cell products for downstream evaluation in clinical trials. iPSC-NK cells can be fine-tuned by multiple genetic modifications to achieve more potent effector function, introduce tailored specificities, and promote persistence, and they are therefore attractive candidates for off-the-shelf cancer immunotherapies [25].

## 5. Conclusions

Sarcomas are malignant tumors with poor prognosis. NK cells have a critical role in controlling every phase of the disease, from the early initiation to the metastatic spread. Hence, the breakdown of NK cell-mediated immunosurveillance unleashes the deadly potential of the disease. Immunoevasion mechanisms of sarcomas include a variety of molecular mechanisms such as MHC class I upregulation, shedding of NK activating ligands, altered oxygen metabolism, and increased expression of inhibitory molecules and cytokines. All of the above-mentioned factors contribute to creating a hostile tumor microenvironment, ultimately leading to NK cell suppression, cancer immunoevasion, and subsequent disease progression. Apart from crucial role of NK cells in sarcoma immunosurveillance, other cell types such as NKT cells have also been shown to control sarcoma growth [38,69,248].

Current therapies have shown a limited capacity for improving the survival of patients with advanced disease. Due to the established role of NK cells in sarcoma development and its intrinsic sensitivity to NK cell lysis, sarcomas are a promising target for therapies utilizing NK cells. Augmenting NK cell anticancer properties can be achieved through various priming strategies and genetic modifications, which improve cancer cell recognition, tumor homing, and resistance to suppressive factors in the TME. Further, sarcoma cells can be sensitized to NK cell-mediated cytotoxicity by monoclonal antibodies, radiotherapy, hyperthermia, HDAC inhibitors, and other treatments. Because of complex multifactorial tumor immunoevasion mechanisms, a combination of both types of strategies is likely needed for a successful treatment outcome. 

The challenge remains in translating current basic research into novel therapies for sarcoma. Initial NK cell-based therapies have shown promising results in a subset of patients, encouraging further clinical trials. One key task is to implement new insights into the functional diversification of NK cells in terms of refined strategies to expand specific subsets, induce memory-like NK cells, and harness NKG2A or KIR-driven education. Another utterly important task is the identification of factors predicting response to NK cell therapy in patients. Despite all the recent advances, further basic research and clinical studies have to be performed to deepen our understanding of NK cell function in the context of sarcoma immunosurveillance, allowing for a knowledge-driven design of sarcoma therapies that will fully utilize NK cell antitumor potential.

## Figures and Tables

**Table 1:** A brief summary of natural killer (NK) cell activating and inhibitory receptors.

Receptor	Known Ligands	Molecular Structure	Function
Killer Immunoglobulin-like receptors (KIR)	HLA-A, Bw, C, G	Immunoglobulin Superfamily	Stimulatory (short cytoplasmic tail) or inhibitory (long cytoplasmic tail)
CD16 (FcγRIII)	Fc portion of IgG	Immunoglobulin Superfamily	Stimulatory
CD2 receptor family		Immunoglobulin Superfamily	
2B4 (CD244)	CD48		Stimulatory
DNAM-1 (CD226)	PVR (CD155) and Nectin-2 (CD112)		Stimulatory
NTB-A	Homophilic		Stimulatory
CS1 (CRACC)	Homophilic		Stimulatory
NKG2 receptor family		C-type lectins	
NKG2D	MICA/B, ULBPs		Stimulatory
CD94/NKG2A	HLA-E		Inhibitory
CD94/NKG2C	HLA-E		Stimulatory
CD94/NKG2E	HLA-E		Stimulatory
Natural Cytotoxicity Receptors (NCRs)		Immunoglobulin Superfamily	
NKp30a/b	B7-H6, BAG6		Stimulatory
NKp30c	B7-H6, BAG6		Inhibitory
NKp44	PCNA		Stimulatory
NKp46	Vimentin		Stimulatory
NKp80	AICL		Stimulatory
